# A Dysregulation of the Prolactin/Vasoinhibin Axis Appears to Contribute to Preeclampsia

**DOI:** 10.3389/fendo.2019.00893

**Published:** 2020-01-09

**Authors:** Livia Lenke, Gonzalo Martínez de la Escalera, Carmen Clapp, Thomas Bertsch, Jakob Triebel

**Affiliations:** ^1^Institute for Clinical Chemistry, Laboratory Medicine and Transfusion Medicine, Nuremberg General Hospital & Paracelsus Medical University, Nuremberg, Germany; ^2^Instituto de Neurobiología, Universidad Nacional Autónoma de México (UNAM), Campus UNAM-Juriquilla, Querétaro, Mexico

**Keywords:** vasoinhibin, prolactin, preeclampsia, 16K PRL, PRL, prolactin/vasoinhibin axis, hypertensive pregnancy disorders

## Abstract

Preeclampsia is a hypertensive disorder affecting 3–5% of all pregnancies. The only curative treatment is delivery of the placenta and the pathophysiology is poorly understood. Studies have demonstrated altered levels of antiangiogenic factors in patients with preeclampsia. One such factor is the antiangiogenic and antivasodilatatory peptide hormone vasoinhibin, which is higher in the circulation, urine, and amniotic fluid of women with preeclampsia. Normal pregnancy is characterized by elevated circulating prolactin and placental lactogen levels, both of which can serve as vasoinhibin precursors when they are enzymatically cleaved. A dysregulation of vasoinhibin generation during preeclampsia is indicated by higher vasoinhibin, prolactin, placental lactogen, and vasoinhibin-generating enzymes levels and activity. The present article integrates known vasoinhibin levels, effects, and signaling mechanisms to the clinical characteristics of preeclampsia to substantiate the notion that vasoinhibin dysregulation can be causally linked to the development of preeclampsia. If this view is demonstrated, assessment of vasoinhibin levels and regulation of its activity could help estimate the risk of preeclampsia and improve its treatment.

## Introduction

Preeclampsia (PE) is a severe complication of pregnancy, defined by gestational hypertension and proteinuria after the 20th week of pregnancy (1). Hypertension is defined as blood pressure >140/90 mmHg in mild PE and >160/110 in severe presentations. Proteinuria is defined as more than 0.3 or 5 g protein in urine collected for 24 h in mild or severe states, respectively (2, 3). In about one of five cases, PE is associated with small-for-gestational-age (SGA) neonates (birth weight below the 10th percentile of the gestational age) (4). The worldwide incidence of PE is described as 3–5% of all pregnancies (5). Beside hypertension and proteinuria, patients may present edema, headache, right upper quadrant or epigastric pain, blurred vision, nausea, and shortness of breath due to pulmonary edema (6). Treatment options are limited since there is no curative therapy except delivery of the placenta. Complications of PE can lead to more severe diseases like eclampsia, characterized by generalized seizure attacks not attributed to other causes (3, 7), or HELLP syndrome (hemolysis, elevated liver enzymes, and low platelets) (8). Besides hypertension and microalbuminuria, long term complications include the development of ischemic heart disease risk elevation, higher stroke rate, venous thromboembolisms and cardiovascular disease risk elevation (9). Numerous studies have investigated anti-angiogenic and angiogenic factors in the circulation of patients with PE, and it is generally accepted that a dysbalance of these factors may not merely represent an epiphenomenon, but rather have a causal role in the pathophysiology of PE. While the exact pathomechanism for preeclampsia is still unclear, it is known that various pro- and anti-angiogenic factors as well as their receptors, play a key role in the development of preeclampsia, since they regulate placental blood flow and vascular development. One of the most widely studied pro-angiogenic factors is vascular endothelial growth factor (VEGF) and the anti-angiogenic soluble form of the VEGF receptor 1 or soluble fms-like tyrosine kinase 1 (sFlt1 or sVEGFR1) (10). A comparatively less studied factor with vascular effects is the pituitary hormone prolactin (PRL), which is the focus of the present article.

A role of PRL in preeclampsia has first suggested around 1975 by Redman, Horrobin (11, 12), and others, but at the time, the possible contribution of antiangiogenic PRL-fragments was not considered as their vascular actions were discovered later, in 1991 (13) (Table 1). Since then, antiangiogenic fragments of PRL, but also of placental lactogen (PL) and growth hormone (GH) (22, 23), evolved as a the vasoinhibin family, which shares specific structural features, biological effects, and signaling mechanisms (24, 25). Vasoinhibin features a variety of endocrine, paracrine, and autocrine effects (25, 26). It is generated by matrix metalloproteinases (MMP), cathepsin-D, and bone morphogenic protein 1 (BMP-1) which cleave PRL in the region of the long loop connecting the third and fourth alpha helix (27–29). The vascular system is one of its major target tissues and its effects include the inhibition of vasopermeability, angiogenesis, and vasodilation (25). All three vasoinhibin-precursor molecules- PRL, GH, and hPL—show major changes in their circulating levels during normal pregnancy (30). In PE, the changes in circulating hormonal levels are altered compared to normal pregnancy, as are the level and activity of the cleaving enzymes determining the generation. However, the studies addressing vasoinhibin levels in PE, the changing values of their precursors, and the altered levels and activity of the cleaving enzymes have not been analyzed in conjunction with each other and with the altered physiology of the prolactin/vasoinhibin axis, the endocrine system that controls the generation and action of vasoinhibin (31). The present article is an attempt to integrate the various findings pointing to dysregulation of vasoinhibin generation and action as contributors to the development of PE (19).

**Table 1:** Selected original research articles and reviews supporting the involvement of the prolactin/vasoinhibin axis in preeclampsia.

**Brief description**	**Year**	**References**
**Original research articles**		
Point mutations in vasoinhibin precursors might impact vasoinhibin generation in PE	2017	(14)
Circulating vasoinhibin alters blood pressure in mice	2016	(15)
Higher circulating vasoinhibin levels and higher activity of cathepsin D in sera from patients with PE	2015	(16)
Urinary PRL concentrations and the presence of vasoinhibin are associated with the severity of PE	2013 2008	(17, 18)
Vasoinhibin is increased in the circulation, urine, and amniotic fluid of preeclamptic women and may contribute to endothelial cell dysfunction and low birth weight	2007	(19)
Plasma PRL levels are raised in pregnant women with pre-eclamptic features	1975	(11)
**Reviews/Hypotheses**		
Hypothesis that vasoinhibin generation by matrix metalloproteases at the feto-maternal interface may contribute to PE	2010	(20)
Hypothesis that excessive placental vasoinhibin generation may contribute to PE	2002	(21)
Hypothesis that prolactin might be involved in the aetiopathology of PE	1975	(12)

## The Prolactin/Vasoinhibin Axis in Pregnancy and Preeclampsia

### The Levels of the Vasoinhibin Precursors, PRL, hPL and GH, Are Elevated in PE

Due to the rise of vasoinhibin precursor levels, the state of pregnancy as such is a predisposition for a dysregulation of the prolactin/vasoinhibin axis. Vasoinhibin-generating enzymes may utilize a manifold higher supply of substrates, and their activity and regulators in the tissue microenvironment would control both, their normal pregnancy-adapted vasoinhibin levels, as well as the pathological (suppressed or excessive) vasoinhibin levels. Circulating PRL levels start to rise in pregnant women at week 10 and reach up to ≅ 200 ng/ml at week 40 of pregnancy (32). At 1 week postpartum, PRL levels in serum are only about 50 ng/ml and return to baseline levels 4–6 weeks after birth. The pregnancy rise in PRL levels is physiological and occurs to prepare for lactation. Remarkably, women with PE demonstrate higher serum and urine levels of PRL, compared to women with normal pregnancy. Compared to normal PRL levels (about 139 ng/ml), mild PE (162.6 ng/ml), and severe PE patients (190.5 ng/ml) show higher PRL levels in serum before induction of labor or cesarean delivery (17), although other studies did not report such findings (19, 33). Similarly, PRL median levels in urine of women with PE are elevated (195.3, 1,342, and 9,830 pg/ml for normal pregnancy, mild, and severe PE, respectively) (17). Of note, the synciotrophoblast secretes hPL [3] and the concentration of PL in serum of pregnant women is detectable at week 5 and rises continuously to very high levels (≅ 4 μg/ml) at the end of pregnancy (34). One week after term, PL level is very low or already undetectable (34). The circulating levels of pituitary growth hormone decline during pregnancy in response to the rise of placental GH (PGH, also named GH-2), which demonstrates agonism at the GH-receptor. PGH levels can be detected after the week 5 of gestation and continually rise until it becomes dominant over the pituitary isoform during the second half of pregnancy (35–37). PGH levels are about 12 ng/ml in normal and 23 ng/ml in preeclamptic pregnancies (38). Hence, all three hormone vasoinhibin pecursors are higher in PE.

### The Levels of Vasoinhibin and Vasoinhibin Generating Enzymes Are Elevated in PE

Vasoinhibin derived from PRL is elevated in serum, urine, and amniotic fluid of women with PE (18, 19). It appears to be generated locally *in utero* placental tissue through the action of various cleaving enzymes such as cathepsin-D. The incubation of PRL with lysates from placental trophoblasts results in its conversion to vasoinhibin, and addition of pepstatin A, a cathepsin D inhibitor, abolishes such conversion. The generation of vasoinhibin is significantly higher when lysates from women with PE are used, compared to lysates from normal pregnant women, indicating a higher activity of cathepsin D in the placenta during PE (19). The detection rate of vasoinhibin in urine occurs in proportional manner to the severity of preeclampsia and can also be demonstrated in other pregnancy complications such as placental abruption, pulmonary edema, and renal failure (17). About 21% of urine samples of women with severe PE demonstrate the presence of vasoinhibin (14 and 16 kDa isoforms) (17). In patients with HELLP syndrome, vasoinhibin appears in about 60% of urine samples (18). The activity of the vasoinhibin-generating enzyme cathepsin D is elevated in serum of patients with PE compared to normal pregnancy (16). During pregnancy, CD activity is about 156%, compared to non-pregnant women, whereas preeclamptic values are around 183%. Soon after delivery this difference is even more pronounced (129 vs. 217%) and still maintained 1 month after (102 vs. 197%) (16). Similar to this observation, MMP serum levels change during pregnancy. MMP-2 and MMP 3 levels are elevated in sera of pregnant women, although circulating MMP-9 level is lower than in the absence of pregnancy (39, 40). Also, MMP-2 levels increase in PE amniotic fluid (40, 41). These data show that both, substrates and enzymes needed for vasoinhibin generation, are higher in pregnancy compared to the normal non-pregnant state. Comparisons between normal pregnancies and PE reveal an even more pronounced elevation of substrate/enzyme levels in patients with PE.

### Mechanistic Implications of Vasoinhibin Actions in PE

VEGF is a major promoter of angiogenesis and vasodilation in the placenta, and its actions are partially mediated by the production of endothelium-derived nitric oxide (NO). Decreased levels of VEGF and NO occur in PE and inhibition of placental VEGF action and NO production causes reduced perfusion of the fetoplacental unit, hypertension, proteinuria, and fetal growth restriction, indicating that blockage of VEGF-induced NO production is causal in preeclampsia (42). Vasoinhibin suppress VEGF-induced endothelial NO synthase (eNOS) activity in endothelial cells and an NO donor reverses the vasoinhibin-mediated inhibition of VEGF-induced endothelial cell proliferation and acetylcholine-induced vasodilation (43). Genetically modified mice, which overexpress hepatic PRL and develop an increase in systemic PRL and vasoinhibin levels, show an increase in systemic blood pressure, a reduced ejection fraction of the left ventricle, and a reduction of the thickness of the intraventricular septum and the left ventricular posterior wall. In mice deficient in eNOS no variation in systemic blood pressure is found when PRL and vasoinhibin levels are elevated, indicating that eNOS plays a critical role in vasoinhibin-mediated blood pressure regulation (15).

### A Dysregulation of the Prolactin/Vasoinhibin Axis Appears to Contribute to Preeclampsia

Altogether, a sequence of events throughout pregnancy, from the rise of vasoinhibin-precursors, an upregulation of vasoinhibin-generating enzymes, an excessive vasoinhibin generation in the placenta or elsewhere, high vasoinhibin levels in the maternal circulation and other compartments, and the consequence of PE manifestation, points toward a dysregulation of the prolactin/vasoinhibin axis in PE (Table 2). A causal inference of the role of vasoinhibin in PE is further consolidated when the various reports are evaluated for their agreement with the 2015 Bradford Hill Criteria (48), whereby any criteria is supported by one or more studies (Table 3).

**Table 2:** Proposed mechanistic pathways involved in preeclampsia.

**State of pregnancy**	**Upstream**	**Event**	**Downstream**	**Clinical symptom/pathology**
Rise of vasoinhibin precursors PRL, PL, PGH and vasoinhibin (17, 32, 34, 35, 44)	Upregulation of vasoinhibin-generating enzymes cathepsin-D, MMP-2, MMP-3 (16, 45) by unknown triggers and predisposing factors such as PRL point mutations (46)	Excessive generation of PRL-, PL-, and PGH-derived vasoinhibin isoforms in the placenta (19, 47)	Entering of placental vasoinhibin into the maternal circulation and into amniotic fluid (17–19)	Reduced placental neovascularization, vasopermeability, and vasodilation, high maternal blood pressure, low birth weight (13, 15, 43)

**Table 3:** Causal inference between vasoinhibin dysregulation and preeclampsia: application of the 2015 Bradford Hill criteria (48).

**Criteria**	**Selected data**	**References**
Strength of association	- The inhibition of VEGF-induced proliferation of endothelial cells by preeclamptic amniotic fluid (AF) correlates directly with the levels of PRL and vasoinhibin (*r* = 0.599, *p* = < 0.05) - Birth weight inversely correlates with PRL and vasoinhibin concentration in preeclamptic AF (*r* = −0.576, *p* = < 0.05) - Serum PRL is higher in gestational hypertension (*p* = 0.032) in mild PE (0.027) and in severe PE (0.006) compared to normal pregnancy	(19) (17)
Consistency	- Clinical and experimental (*in vitro* and *in vivo*) studies, as well as translational studies support a causal role of vasoinhibin in preeclampsia	(15, 17–19)
Specificity	- Vasoinhibin increase in the circulation of mice leads to higher blood pressure, the hallmark of PE, indicative of mechanistic specificity	(15)
Temporality	- Pregnancy is characterized by an increase in vasoinhibin-precursor levels and vasoinhibin in the circulation which precedes the onset of PE - Women who develop PE have impaired endothelium-dependent vasorelaxation and subtle increases in blood pressure	(44, 49)
Biological gradient	- Vasoinhibin is detected in 0% of patients with mild preeclampsia (*n* = 122), 11% of patients with severe preeclampsia (*n* = 261), and 54% of patients with eclampsia and HELLP-syndrome (*n* = 118)	(18)
Plausibility/coherence	- The placenta cleaves PRL to generate vasoinhibin - The activity of PRL cleaving protease, cathepsin D, in serum is higher in patients with PE	(47)
Experiment	- Serum taken from preeclamptic women causes an endothelial cell dysfunction consistent with the biological effects of vasoinhibin - Vasoinhibin increase in the circulation of mice leads to higher blood pressure	(50) (15)
Analogy	- Antiangiogenic factors, such as soluble fms-like tyrosine kinase 1 (sFlt1) and soluble endoglin (sEng) are released by the placenta into maternal blood and cause widespread endothelial dysfunction - Peripartum cardiomyopathy (PPCM), a disease involving the dysregulation of the PRL/vasoinhibin axis, shows a strong epidemiological relationship with PE, which suggests shared pathogenesis	(51)

## Conclusion

Several publications reported a possible involvement of vasoinhibin to the pathophysiology of preeclampsia (Table 1). The majority of studies are associational, however, they include strong mechanistic implications. The present theory is an attempt to integrate the findings reported in these studies addressing measurements of vasoinhibin in biological samples from patients, vascular effects of vasoinhibin, and clinical characteristics of preeclampsia (Table 2). Both, substrate levels and enzyme activity required for vasoinhibin generation, are higher in pregnancy compared to the non-pregnant state. In PE, there is an even more pronounced elevation of vasoinhibin-precursor levels, cleaving enzymes, and vasoinhibin. Increased vasoinhibin production and its antiangiogenic activity imply that elevated levels may be causally involved in PE, for example by contributing to the development of hypertension (Table 2).

The present analysis reinforces the recommendation that a detection of vasoinhibin levels in blood, urine and amniotic fluid can be useful for screening of PE onset, and as a prognostic marker when PE is manifested (19). Also, placental tissue from women with and without PE should be investigated for vasoinhibin, its precursors and vasoinhibin-generating enzymes to validate that the placenta is origin of vasoinhibin which may not only act locally, but also enter the maternal circulation. These implications underscore the need to develop a quantitative assay for vasoinhibin in body fluids that is suitable for routine clinical use, and to improve detection methods to assess vasoinhibin in human tissues. The question of which vasoinhibin isoforms are generated in the placenta, and whether they are generated by cleavage of PRL, PL, PGH, or all three of them, including their overall quantitative composition, requires particular attention.

Further consolidation and clinical evaluation will demonstrate whether the assessment of vasoinhibin levels, its precursors and regulatory enzymes can contribute to estimate the risk of preeclampsia or to improve its treatment.

## Author Contributions

LL and JT wrote the draft of the manuscript. GM, CC, and TB contributed critical revisions and intellectual content. All authors approved the final version of the manuscript.

### Conflict of Interest

The authors declare that the research was conducted in the absence of any commercial or financial relationships that could be construed as a potential conflict of interest.
